# Hydrogen sulphide inhalational toxicity at a petroleum refinery in Sri Lanka: a case series of seven survivors following an industrial accident and a brief review of medical literature

**DOI:** 10.1186/1745-6673-8-9

**Published:** 2013-04-11

**Authors:** Mitrakrishnan Chrishan Shivanthan, Harshani Perera, Saroj Jayasinghe, Panduka Karunanayake, Thashi Chang, Sujatha Ruwanpathirana, Nilwala Jayasinghe, Yamini De Silva, Dinushka Jayaweerabandara

**Affiliations:** 1 National Hospital of Sri Lanka, Colombo, Sri Lanka; 2 Department of Clinical Medicine, Faculty of Medicine, University of Colombo, Colombo, Sri Lanka

**Keywords:** Hydrogen Sulfide/Sulphide, H_2_S, Petroleum, Inhalation, Toxicity, Industrial accident

## Abstract

**Abstract:**

This case series details clinical observations in 7 survivors of accidental hydrogen sulphide inhalation toxicity at a petroleum refinery in Sri Lanka. One survivor developed status epilepticus and severe neurotoxicity whilst another survivor developed delayed respiratory failure; both patients required intensive care management. One victim manifested mild bronchospasms in the immediate post-exposure period and another developed mild perioral numbness 2 days following the exposure. A brief literature review explores the manifestations, pathophysiology and available modalities of treatment of hydrogen sulphide inhalation toxicity.

**Background:**

Hydrogen sulphide (H_2_S) is a highly toxic gas. Accidental deaths following H_2_S exposure is a known hazard amongst petroleum workers exposed to by-products of refineries. Toxicity results mainly due to cellular respiratory poisoning which impairs oxidative phosphorylation. The heart, brain and the lungs are the organs most commonly affected in H_2_S inhalational toxicity leading to varied clinical presentations.

## Case series presentation

Workmen engaged in repair work at an oil refinery in Sri Lanka were accidentally exposed to toxic fumes following a leak in a pipeline in mid-November 2012.

Hydrogen sulphide is the primary established noxious effluent carried in the damaged pipes. Other toxic agents have never been identified during prior analysis of effluent gases by the chemical engineering department of the institution and therefore are either absent or of negligible levels. The victims were exposed to hydrogen sulphide fumes for a period of approximately 10 minutes before rescue was attempted. The concentration of hydrogen sulphide in the vicinity of the accident or levels each individual was exposed to were not measured.

The victims were immediately taken to hospital but two were declared dead on admission. This case series documents the clinical and laboratory observations in 7 of the workmen who survived toxic exposure to H_2_S inhalation. Blood hydrogen sulphide levels could not be measured in the victims as the test is not available in the health sector of Sri Lanka.

### Case 1

A 36-y-old male admitted to the emergency treatment unit within 1 hour of H_2_S exposure was found to be unconscious with cyanosis, labored-breathing, bronchospasms and generalized muscle twitching. He developed three genreralized tonic-clonic convulsions within a period of 30 minutes. His arterial blood oxygen saturation on pulse oximetry was 80% breathing air, while his blood pressure was 80/50 mm Hg. He was resuscitated with bag and mask ventilation with high flow oxygen and nebulized with salbutamol and ipratropium bromide. He developed frequent ventricular ectopics and runs of bradycardia associated with hypotension, but these responded to anti-arrhythmic drugs. Seizures were treated with parenteral benzodiazepines. Additionally, he was given a single dose of intravenous hydrocortisone 200 mg. Capillary plasma glucose was normal. The arterial blood gas analysis during ambu ventilation was found to be normal.

The specific antidote sodium nitrite was administered within 15 minutes of admission, which was 90 minutes post-exposure. Despite these measures, he developed recurrent seizures, deepening unconsciousness and decerebrate posturing. He was treated with parenteral barbiturates, intubated and artificially ventilated with 100% oxygen.

He was noted to have multiple neurofibromas on his body and his medical records confirmed a diagnosis of neurofibromatosis with a history of epilepsy since childhood. He had been seizure-free for almost 5 years following supervised withdrawal of phenytoin sodium in 2008. He had had decompressive-neurosurgery in 2008 for cervical radiculo-myelopathy secondary to neurofibromatosis. He was commenced on phenytoin sodium and sodium valproate via the nasogastric tube in view of recurrent decerebrate posturing. His pupils remained pin point and reacted sluggishly to light. The plantar reflexes were flexor. There were no focal neurological signs. His lungs remained clear except for a few scattered wheezes and he was in sinus rhythm.

By day 3 he had regained consciousness and was extubated uneventfully. However, he had marked truncal ataxia and a scanning dysarthria on recovery. These were associated with dysdiadochokinesia, dysmetria and intention tremor, but sans nystagmus. He also complained of a mild left-sided hearing impairment of recent onset.

Routine haematological and biochemical investigations including full blood count, liver function tests, renal profile and inflammatory markers did not demonstrate any abnormality.

An MRI of the brain done on day 5 failed to detect any structural abnormality to account for the cerebellar dysfunction and hearing loss. A pure tone audiogram done on day 14 was normal.

His anti-epileptic drugs were reduced to phenytoin monotherapy. His dysarthria and ataxia improved over the following two weeks. He was discharged 20 days after admission with residual mild-dysarthria and mild-ataxia of a modified Rankin scale of 2. He continued to have retrograde amnesia of events preceding the occupational accident.

### Case 2

A 46-y-old male exposed to hydrogen sulphide fumes while rescuing his colleagues presented on day 4 post-exposure with worsening shortness of breath, dry cough and noisy breathing. On examination, he was mildly dyspnoeic and had scattered wheezes in both lung fields with no crepitations. His arterial blood oxygen saturation on pulse oximetry was 98%. Cardiovascular examination was unremarkable.

He had pre-exisiting bronchial asthma, hypertension and diabetes mellitus. The asthma was controlled with prophylactic daily inhaled corticosteroids and intermittent inhaled salbutamol. He was a smoker (5 pack-years to date).

A baseline chest radiograph on admission was unremarkable. He had a neutrophil leukocytosis of 23X10^3^/ul. The other cell lines were normal.

He was administered oral steroids (1/mg/kg), theophylline, co-amoxiclav and nebulized salbutamol and ipratropium bromide. However, over the next 3 days his condition deteriorated and he became very dyspnoeic. His oxygen saturation on air was 88% with an arterial oxygen tension of 60 mmHg; PaCO2 was 26 mmHg; and bicarbonate was normal. ECG did not show any abnormalities. Biochemical investigations apart for hypomagnesemia (0.6 mmol/l) were normal. A review chest radiograph did not show a cause for his acute respiratory decompensation. He was admitted to the intensive care unit and treated with high-flow oxygen, intravenous ceftriaxone, intravenous magnesium sulphate, oral potassium supplements and frequent nebulizations. However, his arterial oxygen saturation further reduced requiring non-invasive continuous positive airway pressure support with an FiO_2_ of 60%. His arterial oxygen saturation improved to 97%. He was then administered 1000 mg of methylprednisolone intravenously per day on 3 consecutive days. On the following day, he developed T wave inversion in inferior and lateral leads of his ECG, but cardiac enzymes and echocardiogram remained normal. He gradually improved over the ensuing days and was weaned off the ventilator day 7 post-admission. A subsequent HRCT of the chest did not show any abnormality. He was discharged from hospital 5 days later, with a diagnosis of late chemical pneumonitis and hypoxic cardiac ischaemia following hydrogen sulphide inhalational toxicity.

### Case 3

A 40-year-old male presented in the immediate post-exposure period with bronchospasms without evidence of hypoxia. He was administered nebulized salbutamol and ipratropium bromide, and intravenous hydrocortisone. His haematological and biochemical tests, ECG, and chest radiograph were normal. His oxygen saturation remained stable. He was discharged 3 days post-exposure without further sequelae.

### Case 4

A previously well male developed peri-oral numbness on day 3 post-exposure. He had not had features of acute toxicity. General and systemic examinations were normal. Chvostek and Trousseau signs were both negative. Biochemical tests including serum ionized calcium, magnesium, routine haematological tests, chest radiograph and ECG were normal. He was discharged 2 days following admission.

### Case 5 to 7

Three exposure victims did not develop any clinical or laboratory features of toxicity. They had inhaled only small amounts of the leaked H_2_S fumes during the occupational accident.

## Discussion

Hydrogen Sulphide (H_2_S) is a highly toxic, colorless gas that is recognized by a pungent odour reminiscent of ‘rotten eggs’. It is used in several industries and is a product of many industrial processes such as oil refining, mining and rayon manufacture [1]. H_2_S originates mainly during breakdown of organic matter under anaerobic conditions. Following inhalation, H_2_S dissociates into free sulphide and hydrogen ions in the blood circulation. Sulphide binds to many macromolecules, including cytochrome oxidase thereby preventing oxidative phosphorylation. This causes reversible inhibition of aerobic metabolism leading to cellular anoxia [2,3].

Accidental deaths following accidental H_2_S exposure is a known hazard amongst petroleum workers [4]. H_2_S has also been implicated in deaths in farms following slurry or manure related accidents [5].

The development of seizures in case 1 is of considerable interest. It could be due to the seizure threshold being reached due to irritant effects of anoxia or due to effects of H_2_S itself. H_2_S has been known to characteristically cause sudden loss of consciousness which is colloquially termed 'knockdown' [3,6]. Coma is often associated with severe poisonings by H_2_S [7]. Recent studies have shown that neurons have relatively high H_2_S levels that act as messengers and therefore H_2_S is a neuromodulator [8]. Animal studies indicate that this neuromodulation includes up-regulation and increased expression of gamma-aminobutyric acid B receptor subunits 1 (GABA(B)R1) and 2 (GABA(B)R2) [9]. However, since GABA has inhibitory effects on neurons, one would not expect H_2_S to reduce the seizure threshold [10]. Therefore our patient probably developed seizures as a consequence of the combination of hypoxic and histotoxic hypoxia in the background of a propensity for seizures, rather than a direct toxic effect of H_2_S.

Post-anoxic neurological sequelae have been reported [6]. Survivors have been reported to exhibit neurobehavioral effects following acute, non-fatal H_2_S intoxication with proven reduction of cognitive capabilities, depressive symptoms and personality changes even though the results of neurological examination and neuroimaging techniques remained unremarkable [1]. A case report detailing the sequelae following H_2_S exposure in a 27-year-old male with Glasgow Coma Score which improved from 3 to 15 over a 7 day period with hyperbaric oxygen therapy showed that though both CT and MRI of brain were unremarkable, PET scan 3 years later showed abnormally decreased metabolism bilaterally in the temporal, inferior parietal lobes, left thalamus and abnormal uptake in the striatum. Further cerebral perfusion studies showed bilaterally decreased flow in the putamen without cortical abnormalities. Neuropsychological and neurofunctional testing revealed microsmia, psychomotor slowing, extrapyramidal signs and deficits in memory and executive/planning functioning [11]. The primary effects of H_2_S may be confounded by anoxia or head trauma [3]. Persistent vegetative state is also a described phenomenon [6]. Subclinical and clinical neuropsychiatric disorders have been documented to manifest in the long-term in cases with exposure without acute loss of consciousness [12]. A recent study carried out in mice however demonstrated that when first put into a suspended animation-like state by a 20-min pretreatment with H_2_S followed by exposure to a hypoxic environment survival is prolonged with no apparent detrimental effects compared to mice which were not pretreated with H_2_S. Hence H_2_S also has a propensity to ameliorate hypoxic damage following exposure [13] Studies have also demonstrated that H_2_S can induce a hypometabolic, hibernation-like state in mammals when given in subtoxic concentrations which will reduce the demand for oxygen and minimize unavoidable hypoxia-induced injury such as ischemia/reperfusion injury during renal transplantation [14].

The features of central nervous system toxicity observed in the patient 1 overlaps with previously documented features and pathophysiology of neurotoxicity seen after hydrogen sulphide inhalation which are described above. New onset cerebellar dysfunction though unique to this patient could probably be explained by cytotoxic hypoxic injury to neurons. Despite the pre-existing neurofibromatosis and past history of seizures controlled by antiepileptics the temporal relationship to hydrogen sulphide exposure clearly establishes a cause and effect link between the abnormality and the toxic agent. PET scanning was not performed on our patient due to financial constraints despite literature evidence of studies showing abnormalities on PET imaging which were undetectable on MRI and CT scanning. Early administration of antidote and ventilation with 100% oxygen is likely to have minimized the severity of hypoxic damage. Persistent retrograde amnesia could either a post traumatic event or more likely the result of hypoxic damage to neurons involved in the complex tasks of processing memories.

The delayed onset respiratory failure seen in patient 2 is probably explained by a reactive airway dysfunction syndrome for which he probably could have had a predisposition as a result of asthma and chronic smoking. The absence of radiological evidence does not disprove the entity as the pathology is one of airways rather than gas exchange interface or alveolar parenchyma. Response to steroids and ventilator support with high FiO2 with complete reversibility and the temporal relationship to the exposure in this instance establish the relationship between the phenomenon and the toxic agent. Hypomagnesemia is a new observation hitherto unreported following hydrogen sulphide inhalation and is worthwhile looking for in future cases. Late onset perioral numbness reported in one of the cases was not substantiated by other clinical features of hypocalcemia or biochemical investigations to suggest a disturbance of calcium or magnesium homeostasis.

Animal studies following H_2_S poisoning have demonstrated respiratory and circulatory changes as early as 10–15 minutes with 100% involvement in 20–25 minutes in anaesthetized cats with severe respiratory disturbances and abrupt decrease of mean systemic arterial pressure and decreased cardiac output [15]. Studies in rats have demonstrated morpho-functional alterations in the aero-hemic barrier and surfactant system of the lungs with resultant pulmonary oedema [16]. Acute respiratory failure caused by an acute pulmonary oedema with left ventricular systolic dysfunction as a possible cause of this cardiorespiratory complication has been purported in medical literature [7]. Exposure to higher concentrations increase the risk of pulmonary oedema [3]. A late onset reactive airways dysfunction syndrome (RADS) after exposure to a high level of toxic gases mainly hydrogen sulphide has been described in a previously non-atopic man which had manifested with moderate, partially reversible, airway obstruction and increased responsiveness [17]. Hemorrhagic bronchitis and pneumonitis have been demonstrated in postmortem studies and subacute lung injuries following long term exposure to hydrogen sulphide has also been postulated [18].

Hydrogen sulphide induced mucosal irritation may predominate at lower concentrations with a keratoconjunctivitis called 'gas eye' [3] Animal model studies using exfoliative cytology has yielded quantitative information of value in ocular irritation studies [19].

Blood sulphide levels are useful in the diagnosis of hydrogen sulphide toxicity and help to differentiate it from others with a similar presentation. Delta aminolaevulunic acid synthase and haem synthase levels are known to be decreased following exposure to hydrogen sulphide; however no correlate could be drawn to levels of blood sulphide in survivors [20].

Treatment following hydrogen sulphide toxicity is currently empirical, with a combination of nitrite and hyperbaric oxygen [3,21,22]. 4-dimethylaminophenol has also administered as an antidote, followed immediately by hyperbaric oxygen therapy [2].

The use of hyperbaric oxygen therapy (HBOT) for H_2_S poisoning remains controversial, but has a similar underlying rationale to that in carbon monoxide poisoning. HBOT may play a useful role in improving oxygenation and acid–base status quickly and counteracting the decrement in oxygen carriage caused by methaemoglobinaemia due to antidote administration [2].

## Conclusions

The authors recommend measures to improve safety of workers to minimize inhalational toxicity in situations with possible hydrogen sulphide exposure. Vigilant reporting of features of toxicity is needed in order to collate the features of toxicity of this noxious gas. Antidote use and hyperbaric oxygen therapy is beneficial in cases with severe exposure and significant toxicity and probably minimizes hypoxic tissue damage especially in the central nervous system.

## Consent

Written informed consent was obtained from the patients for publication of this Case report and any accompanying images. A copy of the written consent is available for review by the Editor-in-Chief of this journal.

## Competing interests

The authors declare that they have no competing interests.

## Authors’ contributions

All the authors were involved in the clinical management of the cases described, drafting of the manuscript, and literature survey. All authors read and approved the final manuscript.
